# The Relationship between the Incidence of Postoperative Cognitive Dysfunction and Intraoperative Regional Cerebral Oxygen Saturation after Cardiovascular Surgery: A Systematic Review and Meta-Analysis of Randomized Controlled Trials

**DOI:** 10.31083/j.rcm2312388

**Published:** 2022-11-28

**Authors:** Luchen Wang, Zekun Lang, Haoyu Gao, Yanxiang Liu, Huishu Dong, Xiaogang Sun

**Affiliations:** ^1^Aortic and Vascular Surgery Center, Fuwai Hospital, National Center for Cardiovascular Diseases, Chinese Academy of Medical Sciences and Peking Union Medical College, 100037 Beijing, China; ^2^The First Clinical Medical College of Lanzhou University, 730000 Lanzhou, Gansu, China

**Keywords:** postoperative complication, cardiovascular surgery, regional cerebral oxygen saturation (rScO_2_), meta-analysis

## Abstract

**Background:**

To assess whether intraoperative monitoring and intervention of regional cerebral oxygen saturation levels can reduce the incidence of postoperative cognitive dysfunction in patients undergoing cardiovascular surgery and contribute to patient prognosis.

**Methods:**

The Cochrane Library, PubMed, and the Web of Science were systematically searched for relevant randomized controlled trials involving the effects of cerebral oxygen saturation on the cognitive function of patients after cardiovascular surgery from January 1, 2000 to May 1, 2022. The primary outcome was the incidence of postoperative cognitive dysfunction. The secondary outcomes were length of hospital stay, length of intensive care unit (ICU) stay, length of mechanical ventilation, length of cardiopulmonary bypass, and other major postoperative outcomes such as renal failure, infection, arrhythmia, hospital mortality, and stroke. Data were pooled using the risk ratio or standardized mean difference with 95% confidence interval (CI). The original study protocol was registered prospectively with PROSPERO (CRD42020178068).

**Results:**

A total of 13 randomized controlled trials involving 1669 cardiovascular surgery patients were included. Compared with the control group, the risk of postoperative cognitive dysfunction was significantly lower in the intervention group (RR = 0.50; 95% CI: 0.30 to 0.85; *p* = 0.01; $I^{2}$ = 71%). The Duration of stay in intensive care units in the intervention group was also significantly shorter than that in the control group (standard mean difference (SMD) = –0.14; 95% CI: –0.26 to –0.01; *p* = 0.03; $I^{2}$ = 26%). Univariate meta-regression analyses showed that age is a major source of heterogeneity.

**Conclusions:**

Our current study suggests that intraoperative cerebral oxygen saturation monitoring and intervention can significantly reduce the incidence of postoperative cognitive dysfunction, and the length of intensive care unit stay after intervention is considerably reduced. Given that some limits in this review, more high-quality, and long-term trials are still needed to certify our findings.

## 1. Introduction

Postoperative cognitive dysfunction (POCD) is a common postoperative complication, manifested mainly as cognitive impairment, memory loss, and executive dysfunction, which can lead to adverse reactions such as prolonged hospital stay and decreased quality of life [1]. Several studies have shown that POCD occurs after various operations, and the incidence of cardiovascular surgery is higher than that of non-cardiac surgery [2]. In recent years, the incidence of POCD in cardiovascular surgery patients has steadily increased, however, due to the lack of understanding of POCD and its risk factors, there is currently no effective prediction and treatment methods [3]. The incidence of POCD in cardiac surgery can also be high, up to 70%, despite optimal oxygen levels [4]. Presently, decreased intraoperative cerebral oxygen saturation has been confirmed to be related to postoperative neurological dysfunction and neurobehavioral deterioration [5]. Therefore, regional cerebral oxygen saturation (${\mathrm{rScO}}_{2}$), as a sensitive indicator of cerebral hypoxia or cerebral ischemia, may be used to predict postoperative brain injury in patients undergoing cardiac surgery.

Near-infrared spectroscopy (NIRS), a noninvasive method for monitoring cerebral oxygen saturation in real-time, is widely used during perioperative procedures [6]. In order to ensure sufficient cerebral perfusion and minimize the risk of these neurocognitive complications, anesthesiologists use cerebral oxygen saturation monitoring [7]. Although cerebral oxygen saturation monitoring has been used as a clinical auxiliary technology for more than 20 years, there is a lack of sufficient consensus on the management of cerebral oxygen saturation during the perioperative period [8]. Some prospective randomized controlled trials have sought to assess the effect of cerebral oxygen saturation on postoperative cognitive outcomes. Nevertheless, the results remain inconclusive [9]. In addition, some randomized controlled studies have confirmed the predictive value of ${\mathrm{rScO}}_{2}$ monitoring for POCD [5, 10, 11].

Studies have shown that in cardiac surgery patients, besides inflammation, cardiac arrest or complicated by sepsis, the reduction of ${\mathrm{rScO}}_{2}$ (60%) is the most relevant factor leading to an increased risk of cognitive dysfunction [12, 13]. On the contrary, some studies believe that for patients undergoing cardiac surgery, whether it is at discharge or 3 months after discharge, there is no difference in the intraoperative ${\mathrm{rScO}}_{2}$ variables between patients with and without cognitive impairment [14]. In addition, it is also of great significance to explore whether NIRS detection and intervention of intraoperative brain oxygen saturation has a positive effect on prognosis and can reduce the economic burden of POCD patients. Therefore, this study aims to explore whether intraoperative ${\mathrm{rScO}}_{2}$ monitoring and treatment can reduce the incidence of POCD in patients undergoing cardiovascular surgery.

## 2. Methods

The study was conducted according to the Preferred Reporting Items for Systematic Reviews and Meta-Analyses Protocols (PRISMA) statement [15]. The protocol has been registered in the International Prospective Systematic Reviews Registry database (CRD42020178068).

### 2.1 Data Sources and Search Strategy

The PubMed, Web of Science and Cochrane Library were searched from January 2000 to May 2022. The related searching words were as follows: [(Cognitive Decline) OR (Cognitive Dysfunction) OR (Cognitive Impairment) OR (Mental Deterioration) OR (Mild Cognitive Impairment) OR (Mild Neurocognitive Disorder)] AND [(Cardiovascular Disease) OR (myocardial infarct) OR (coronary heart disease) (myocardial infarct)] AND (regional cerebral oxygen saturation) in the title/abstract. Moreover, citations from articles were retrieved in order to identify relevant studies that were not included in the initial literature search. A search of clinicaltrials.gov was also conducted to identify any ongoing RCTs with results expected in the near future. The detailed search strategy is presented in the **Supplementary Material** in the form of a word document.

### 2.2 Selection Criteria

Inclusion criteria were pre-specified according to the PICOS approach (See the **Supplementary Table 1** for details). Inclusion criteria: (1) The literature is a randomized controlled trial which must include the intervention group that receives ${\mathrm{rScO}}_{2}$ monitoring and intervention to maintain brain oxygen saturation at a higher level and the control group that does not receive monitoring; (2) participants over 18 years old and have cardiac surgery at the surgical site; (3) the outcome indicators were the incidence of POCD and other typical postoperative complications. Exclusion criteria: (1) reviews, conference abstracts, case reports, and other non-RCT studies; (2) identical or similar repeated report research; (3) literature with incomplete data or the data cannot be converted into usable data. Based on predetermined inclusion and exclusion criteria, two authors (WLC and LZK) reviewed and selected the studies independently. Inconsistencies were addressed through discussion with SXG.

### 2.3 Data Collection and Quality Assessment

Data extraction was performed independently by 2 reviewers (WLC and LZK). Any disagreements were resolved by consensus or by consulting SXG. The data extraction content includes: first author, year of publication, sample size, research type, research object, intervention measures, control measures, primary and secondary outcomes, surgical method, cardiopulmonary bypass time, mechanical ventilation time, number of POCD cases, and major postoperative complications.

According to the “Randomized Trial Bias Risk Assessment Tool” in the Cochrane Handbook, the quality of the included literature is evaluated [16]. The evaluation content includes allocation hiding, randomization method, blinding method between investigator and subject, blinding method of result evaluator, and selective reporting of results, data completeness, and other possible biases in seven areas. The overall risk of bias judgement can be rated as low risk of bias, unclear risk of bias, and high risk of bias [16].

### 2.4 Outcomes and Definitions

The primary outcome was the incidence of POCD. The secondary outcomes were length of postoperative hospital stay and intensive care unit (ICU) stay. Additional outcomes were cardiopulmonary bypass (CPB) time and mechanical ventilation time and major postoperative complications such as stroke, renal failure, infection, arrhythmia, and hospital mortality.

### 2.5 Statistical Analysis

All data analyzed by Review Manager (RevMan) version 5.4 (The Cochrane Collaboration, Copenhagen, Denmark) and Stata SE 16.0 (Stata Corporation, College Station, TX, USA). The risk ratio (RR) with 95% confidence intervals (CI) were estimated for dichotomous data, and standard mean difference (SMD) with 95% CI for continuous data, respectively. In order to take into account methodological and clinical heterogeneity, the fixed and random effect models were employed to assemble the data [16]. The Q-test and $I^{2}$ statistic were used to assess the heterogeneity of studies. Significant heterogeneity was considered when *p *< 0.1 or $I^{2}$ > 50%. Subgroup and meta-regression analysis were conducted to explore the possible source of heterogeneity. Risk of publication bias for studies will be assessed using funnel plots, and the Egger’s test was employed to examine the publication bias when there were at least 10 studies. A significance level of α = 0.05 was set for all analyses. Sensitivity analysis was used to assess whether the results were robust and also to assess sources of heterogeneity.

## 3. Results

### 3.1 Trial Selection

**Fig. 1.**
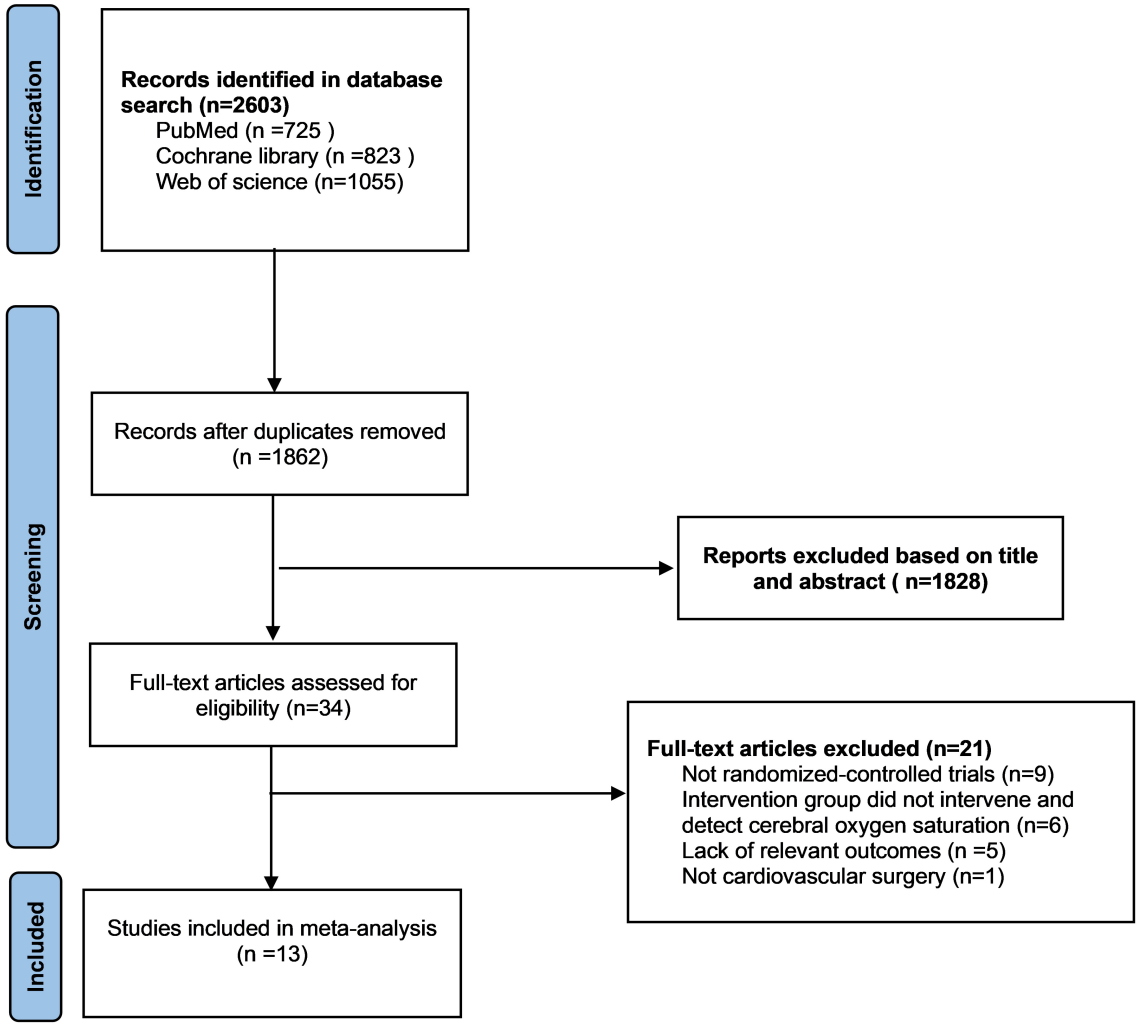
**Preferred reporting items for systematic reviews and meta-analyses (PRISMA) flowchart of selection**.

A total of 2603 articles were retrieved from the database, and after deduplication, 1862 article titles and abstracts were evaluated, and then 34 articles were read in full to determine whether they met the criteria for inclusion in the study. Among them, 21 articles were excluded. 9 articles were omitted because they were not randomized controlled trials. 5 articles were excluded because of a lack of relevant outcome indicators. 6 articles were eliminated because cerebral oxygen saturation was not monitored and intervened and 1 article was removed because it’s not cardiovascular surgery (Fig. 1). Thirteen trials were finally included [5, 11, 17, 18, 19, 20, 21, 22, 23, 24, 25, 26, 27].

### 3.2 Study Characteristics and Quality Assessment

**Fig. 2.**
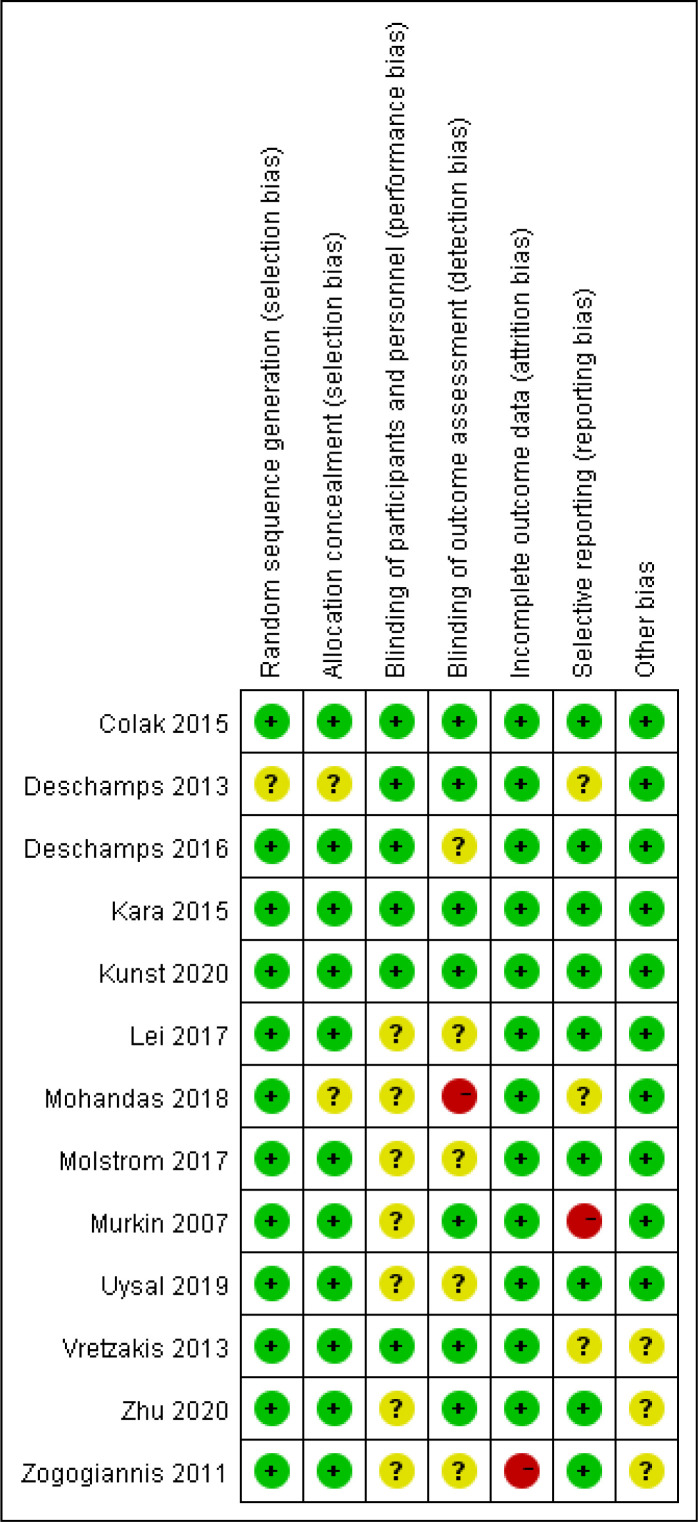
**Risk of bias summary: review authors’ judgements about each risk of bias item for each included study**.

**Fig. 3.**
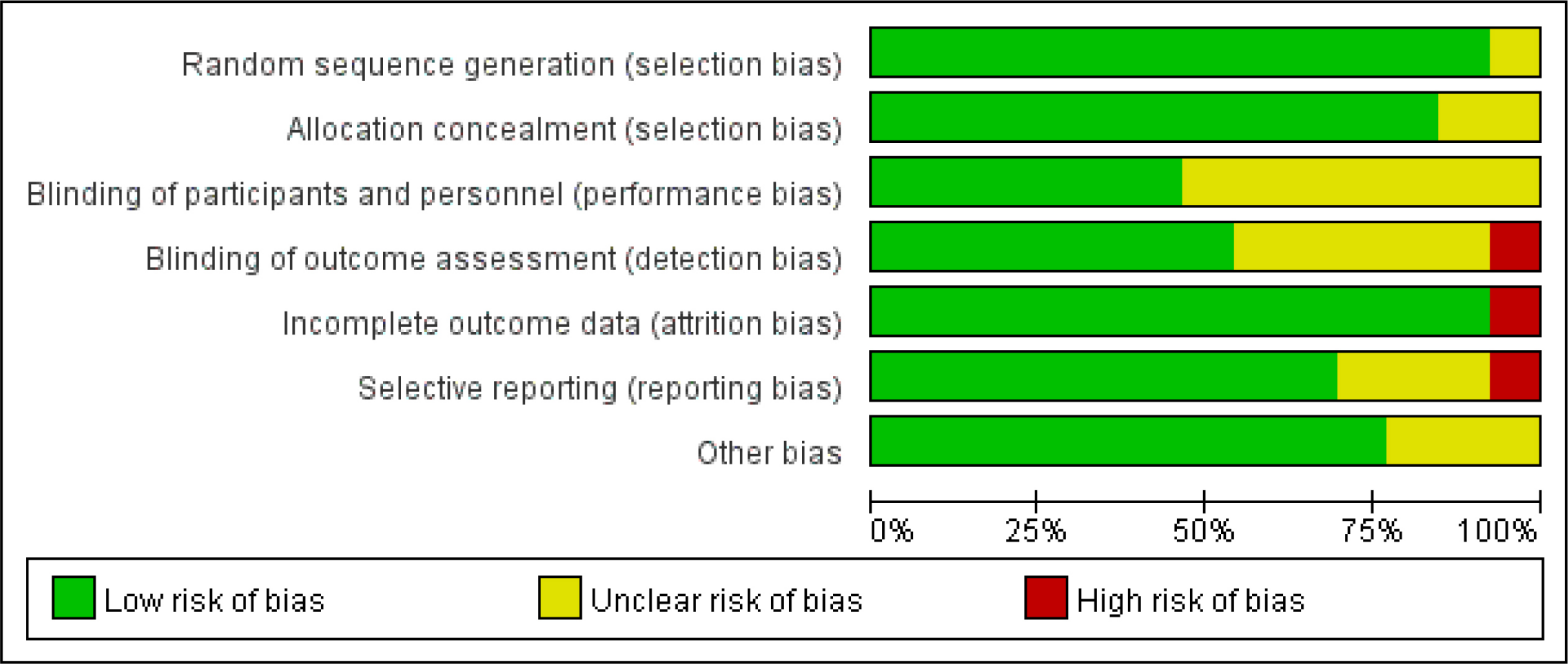
**Risk of bias graph: review authors’ judgements about each risk of bias item presented as percentages across all included studies**.

The basic characteristics of included studies were summarized in Table 1 (Ref. [5, 11, 14, 17, 18, 19, 20, 21, 22, 23, 25, 26, 27]), these trials were reported from 2007 to 2020. Sample sizes for individual experiments ranged from 10 to 249. Table 2 (Ref. [5, 11, 14, 17, 18, 19, 20, 21, 22, 23, 25, 26, 27]) presents the basic information about the patient population included in the study. A total of 1669 patients were involved, and the overall mean age of the patients included in the study was 64.7 years. All patients underwent cardiovascular-related surgery. An overview of the risk of bias assessment for each included trial can be found in Figs. 2,3. As a whole, three trials were classified as having a low risk of bias [5, 19, 20], seven as having an unclear risk of bias [17, 18, 21, 22, 24, 25, 26], and three as having a high risk of bias [11, 23, 27].

**Table 1.** **Baseline characteristics of included studies for meta-analysis**.

References	Country	Sample size		Age		Surgery	Outcome
		Intervention	Control	Intervention	Control		
Colak 2015 [5]	Croatia	94	96	61.9 ± 7.1	63.4 ± 8.8	CABG	POCD incidence, stroke, RF, infection, arrhythmia, MVD, CPB time
Deschamps 2013 [17]	Canada	23	25	71.1 ± 7.9	70.2 ± 9.2	High-risk cardiac surgery	hospital stay, MVD, CPB time
Deschamps 2016 [18]	Canada	102	99	69 ± 12.6	72 ± 9.4	Cardiac surgery	POCD incidence, stroke, ICU time, RF, infection, arrhythmia, death, hospital stay, MVD, CPB time
Kara 2015 [19]	Turkey	43	36	59.1 ± 9.4	61.2 ± 10.3	CABG	POCD incidence, ICU time, hospital stay, CPB time
Kunst 2020 [20]	England	42	40	71.6 ± 5.0	72.0 ± 4.3	CABG	POCD incidence, ICU time, RF, infection, arrhythmia, death, hospital stay, CPB time
Lei 2017 [21]	America	123	126	74.2 ± 6.5	72.9 ± 6.3	Cardiac surgery	POCD incidence, stroke, ICU time, RF, infection, arrhythmia, death, hospital stay, MVD
Mohandas 2013 [11]	India	50	50	34.60 ± 16.28	38.05 ± 15.81	Open heart surgery	POCD incidence, ICU time, MVD, CPB time
Molstrom 2017 [22]	Denmark	5	5	69 [66–72]	72 [72–77]	Cardiac surgery	hospital stay, CPB time
Murkin 2007 [23]	Canada	100	100	61.8 ± 9.3	61.8 ± 10.3	CABG	Stroke, ICU time, RF, infection, arrhythmia, death, hospital stay, MVD, CPB time
Uysal 2019 [14]	America	59	66	57 ± 11	58 ± 12	Cardiac surgery	POCD incidence, ICU time, RF, death, hospital stay, MVD, CPB time
Vretzakis 2013 [25]	America	75	75	67.3 ± 8.5	65.9 ± 9.5	Cardiac surgery	ICU time, RF, arrhythmia, death, hospital stay, MVD, CPB time
Zhu 2020 [26]	China	33	33	61.52 ± 7.97	60.27 ± 9.19	CVR	POCD incidence, CPB time
Zogogiannis 2011 [27]	Greece	83	86	69.1 (50–82)	68.4 (48–81)	Carotid endarterectomy	POCD incidence, stroke, death

POCD, postoperative cognitive dysfunction; CABG, coronary artery bypass graft; RF, renal failure; MVD, mechanical ventilation duration; CVR, cardiac valve replacement; ICU, intensive care unit; CPB, Cardiopulmonary bypass. Data are expressed as mean ± SD or median [IOR] or Mean (range).

**Table 2.** **Baseline characteristics, medical conditions, and perioperative data of included studies for meta-analysis**.

Reference	Age (year)	Male (%)	BMI	Pre-MI (%)	DM (%)	HT (%)	CVA (%)	COPD (%)	CPB duration (min)	Euroscore	Baseline LVEF (%)
Colak 2015 [5]	62.7	78.0	NA	11.6	33.7	90.5	NA	NA	90.0	2.3	56
Deschamps 2013 [17]	70.6	68.8	NA	NA	NA	NA	NA	NA	116.6	NA	56.3
Deschamps 2016 [18]	71.0	72.1	NA	4.5	29.4	79.6	NA	9.9	135.9	5.3	NA
Kara 2015 [19]	60.1	78.5	NA	NA	30.4	74.7	12.7	15.2	78.1	NA	54.0
Kunst 2020 [20]	71.8	81.7	26.7	NA	31.7	92.7	14.6	11.0	81.1	4.4	NA
Lei 2017 [21]	73.5	70.7	28.1	12.4	27.7	76.7	14.5	12.9	111.0	NA	NA
Mohandas 2013 [11]	36.3	58.0	20.7	NA	NA	NA	NA	NA	88.7	NA	NA
Molstrom 2017 [22]	71.5	90.0	25.8	NA	NA	NA	NA	NA	99.6	NA	NA
Murkin 2007 [23]	61.8	87.5	29.6	5	28.5	NA	17.5	17.5	88.2	NA	NA
Uysal 2019 [14]	57.5	68.8	26.8	NA	NA	NA	NA	NA	131.1	2.5	60.0
Vretzakis 2013 [25]	66.6	82.0	27.8	54.7	24	80.6	21.3	21.3	91.3	NA	47.8
Zhu 2020 [26]	60.9	NA	NA	NA	NA	NA	NA	NA	105.3	NA	NA
Zogogiannis 2011 [27]	68.7	69.2	28.0	NA	26.6	53.8	NA	NA	NA	NA	NA

BMI, body mass index; MI, myocardial infarction; DM, diabetes mellitus; HT, hypertension; CVA, cerebrovascular accident; COPD, chronic obstructive pulmonary disease.

### 3.3 Primary Outcomes

**Fig. 4.**
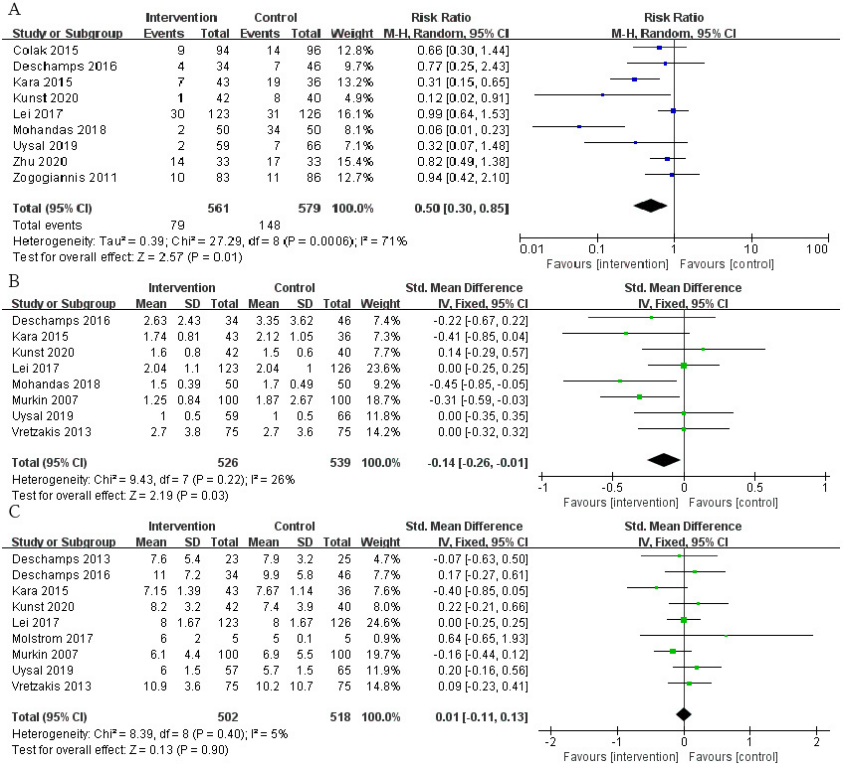
**The pooled effect of (A) postoperative cognitive dysfunction (POCD) incidence. (B)Intensive care unit (ICU) time. (C) Hospital stay**.

The incidence of POCD in a total of 912 patients in 8 RCTs was analyzed. The risk of POCD in the intervention group was significantly lower than that in the control group (Fig. 4A; RR, 0.50; 95% CI: 0.30 to 0.85; *p* = 0.01; $I^{2}$ = 71%).

### 3.4 Secondary Outcomes

A total of 8 RCTs reported patients’ ICU time, involving a total of 1065 patients. The time of ICU stay in the intervention group was shorter, and the difference was statistically significant (Fig. 4B; SMD, –0.14 days; 95% CI: –0.26 to –0.01; *p* = 0.03; $I^{2}$ = 26%). Nevertheless, it was found that the length of hospital stay did not differ significantly between the two groups (Fig. 4C; SMD, 0.01 days; 95% CI: –0.11 to 0.13; *p* = 0.90; $I^{2}$ = 5%).

**Fig. 5.**
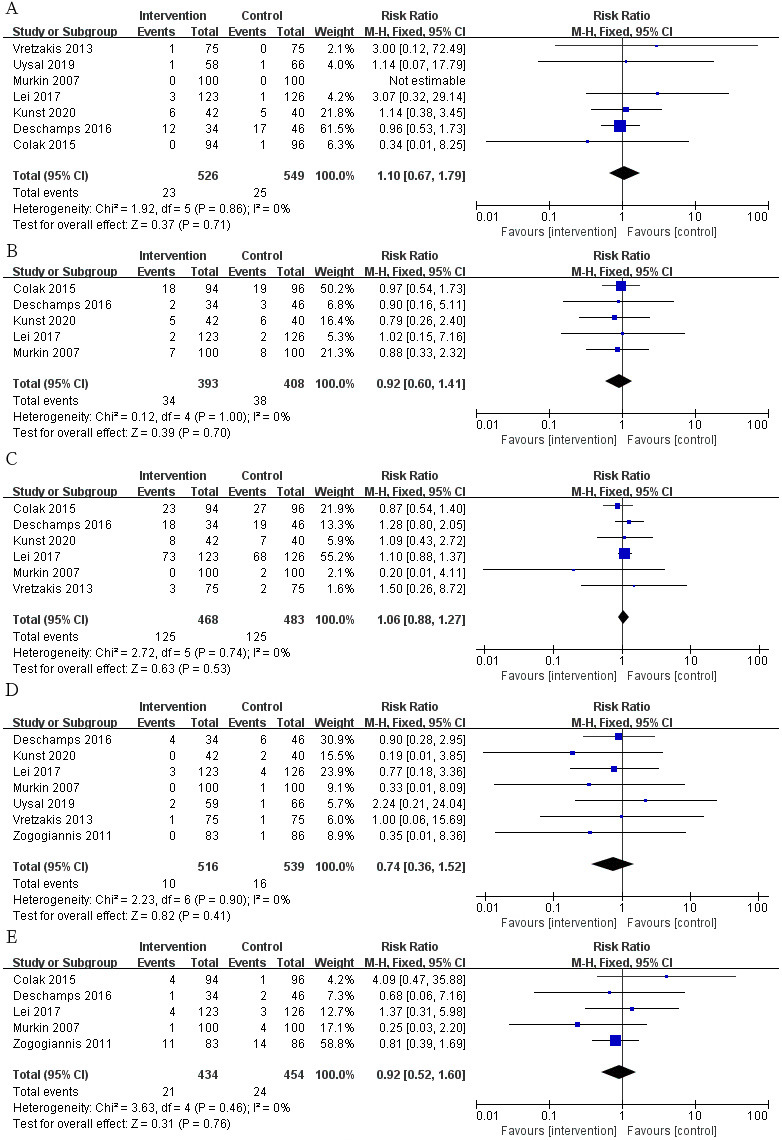
**The pooled effect of (A) renal failure. (B) Infection. (C) Arrhythmia. (D) Hospital mortality. (E) Stroke**.

We also selected some routine postoperative complications as secondary outcomes to evaluate the prognosis of patients, but there were no remarkable differences regarding the incidence of renal failure (Fig. 5A; RR, 1.10; 95% CI: 0.67 to 1.79; *p* = 0.71; $I^{2}$ = 0%), infection (Fig. 5B; RR, 0.92; 95% CI: 0.60 to 1.41; *p* = 0.70; $I^{2}$ = 0%), arrhythmia (Fig. 5C; RR, 1.06; 95% CI: 0.88 to 1.27; *p* = 0.53; $I^{2}$ = 0%), hospital mortality (Fig. 5D; RR, 0.74; 95% CI: 0.36 to 1.52; *p* = 0.41; $I^{2}$ = 0%) and stroke (Fig. 5E; RR, 0.92; 95% CI: 0.52 to 1.60; *p* = 0.76; $I^{2}$ = 0%) between the two groups.

**Fig. 6.**
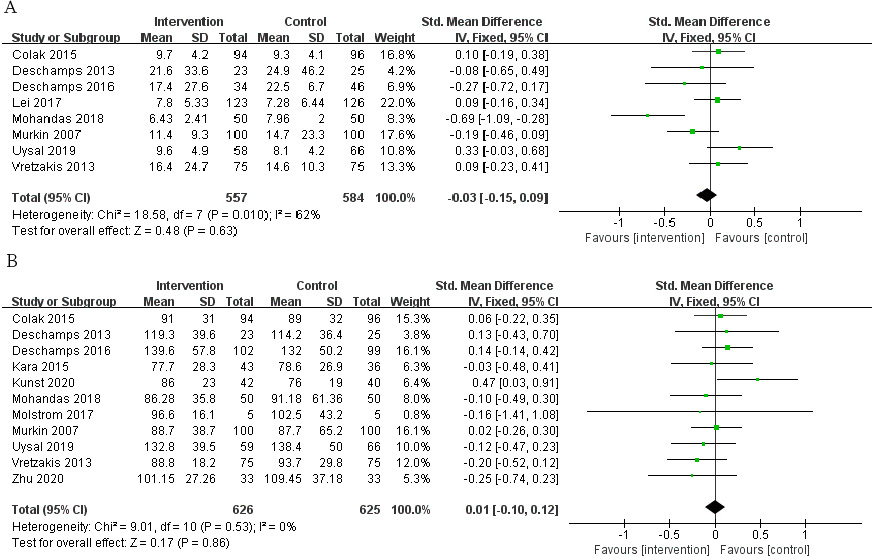
**The pooled effect of (A) mechanical ventilation duration. (B) Cardiopulmonary bypass (CPB) time**.

Compared with the control group, the mechanical ventilation duration (Fig. 6A; SMD, –0.03; 95% CI: –0.15 to 0.09; *p* = 0.63; $I^{2}$ = 62%), and CPB time (Fig. 6B; SMD, 0.01; 95% CI: –0.10 to 0.12; *p* = 0.86; $I^{2}$ = 0%) are not significantly different.

### 3.5 Subgroup Analysis

**Fig. 7.**
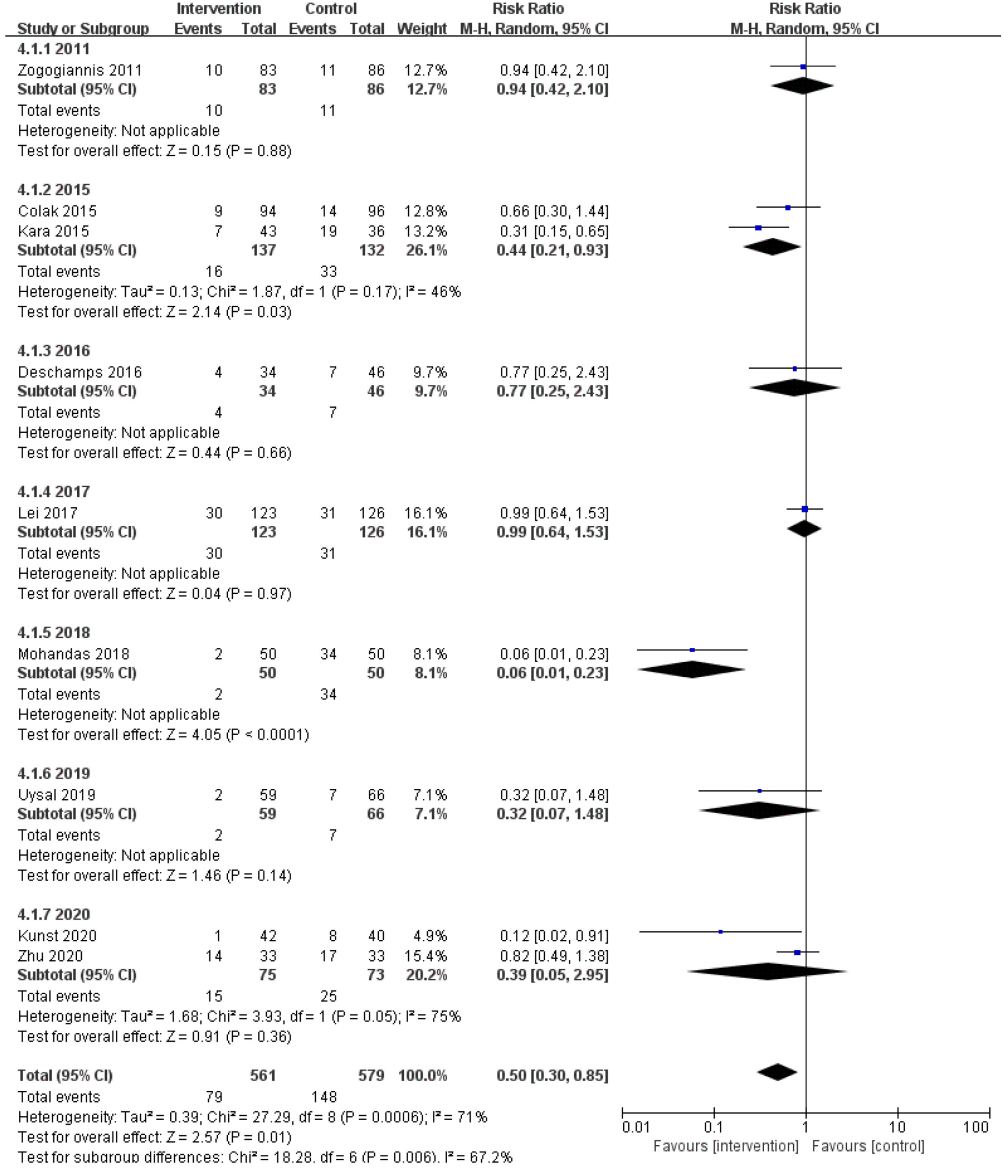
**Subgroup analysis of different publication year groups**.

**Fig. 8.**
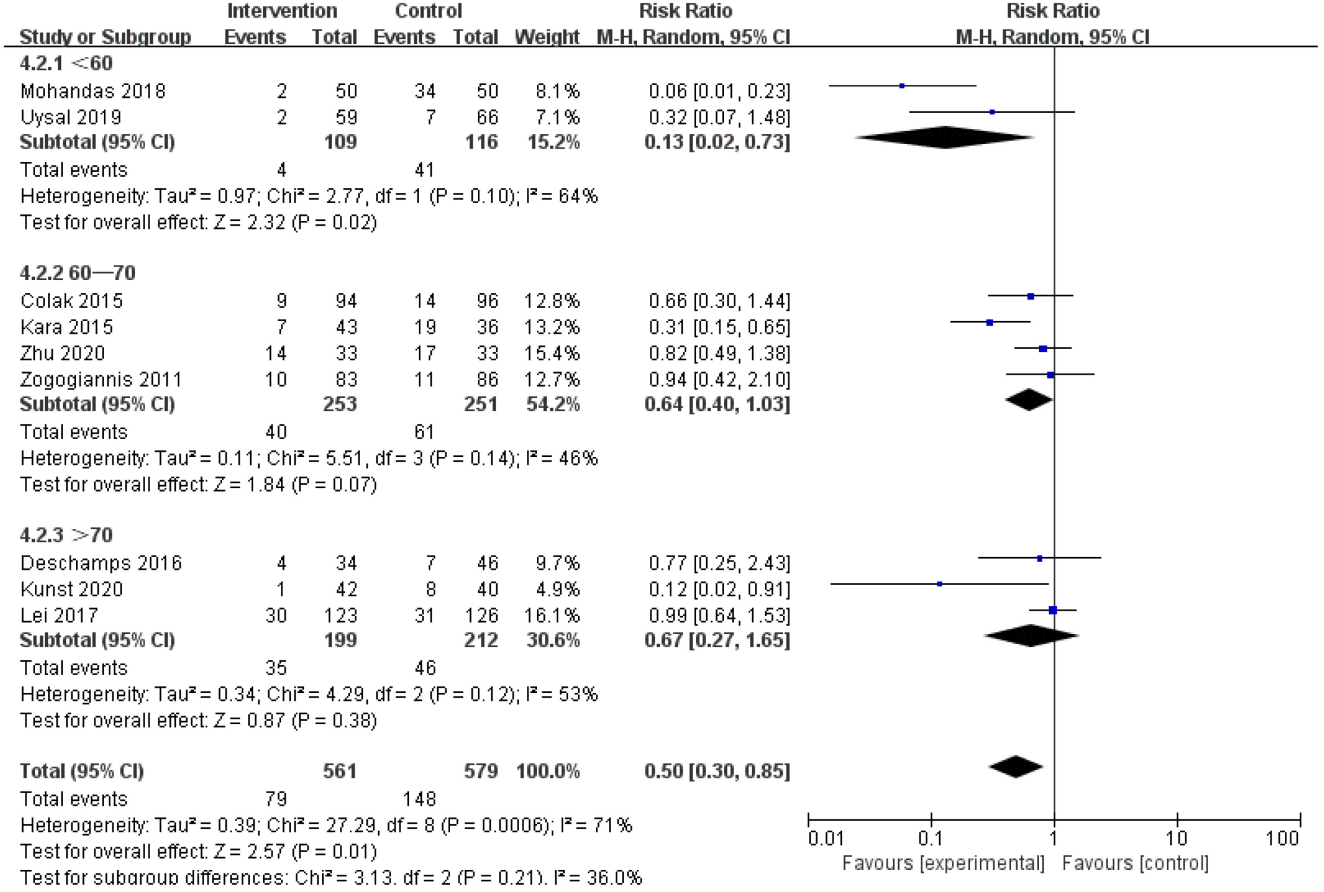
**Subgroup analysis of different age groups**.

**Fig. 9.**
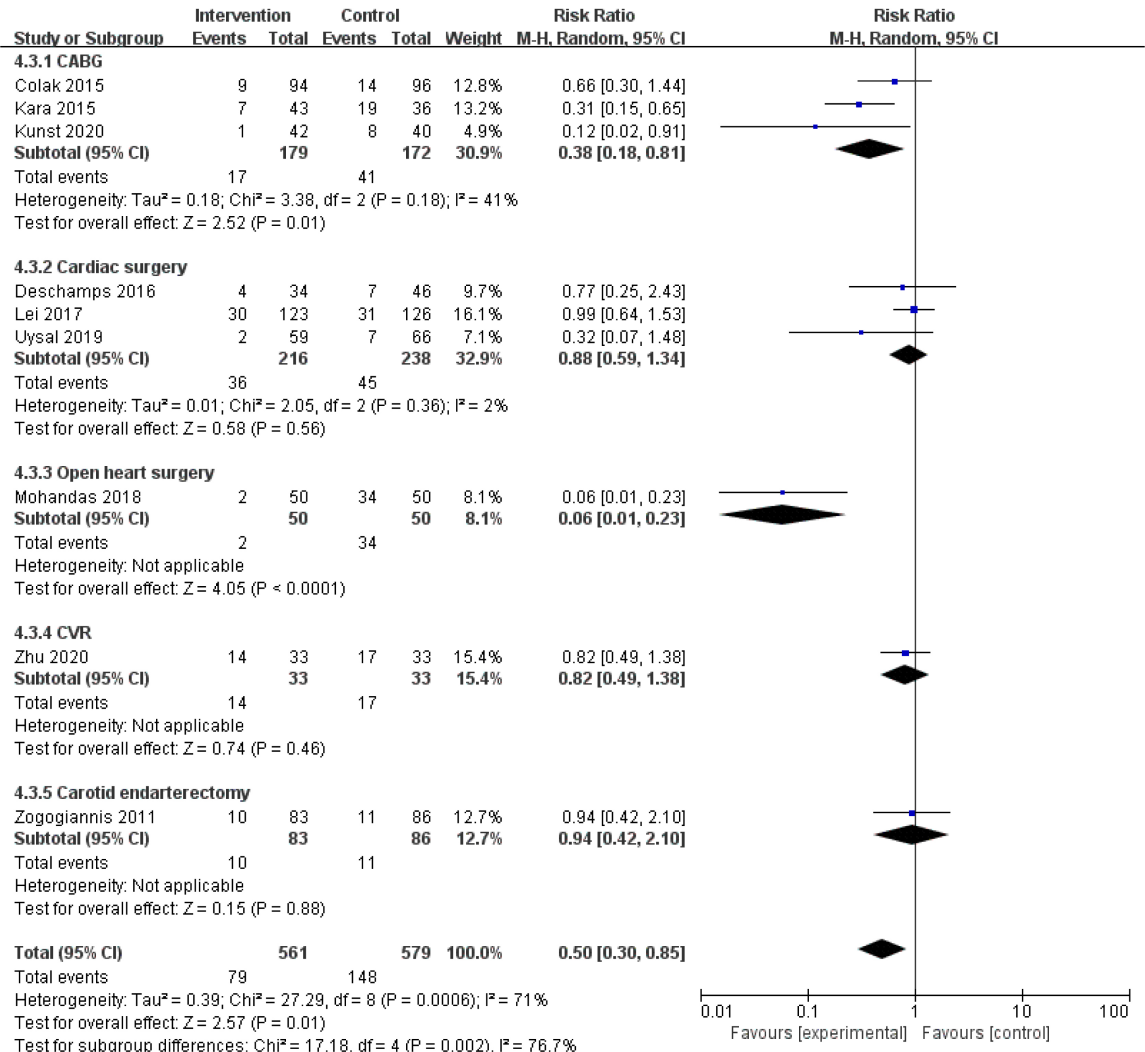
**Subgroup analysis of different surgery types groups**.

We speculated that the year of trial publication year (Fig. 7), the age of the patients (Fig. 8), and the type of surgery (Fig. 9) might be sources of heterogeneity and performed subgroup analyses. Results show lower risk of POCD in people younger than 60 years old.

### 3.6 Meta-Regression for the Potential Sources of Heterogeneity

In the random-effect univariate meta-regression analysis of POCD incidence, variables such as year of publication, age, male, past myocardial infarction, diabetes, hypertension, cerebrovascular accident, chronic obstructive pulmonary disease (COPD), European cardiac operative risk evaluation system, and baseline left ventricular ejection fraction were considered. The main source of heterogeneity in the incidence of POCD is age (coefficient = 0.06; *p* = 0.01; adjusted $R^{2}$ = 0.86).

### 3.7 Publication Bias Assessment and Sensitivity Analysis

The funnel plot of POCD incidence has no obvious asymmetry. Results also showed that there was no apparent publication bias in POCD incidence (Begg’s *p* = 0.08) and ICU time (Begg’s *p* = 0.54). For other outcomes, due to the small number of included studies, no begger test or egger test was performed, but there was no obvious asymmetry in the funnel plot. After excluding the studies of Lei *et al*. [21] and Mohandas *et al*. [11], $I^{2}$ = 35%, the risk of POCD in the intervention group was significantly lower than that in the control group (*p *< 0.05). This is consistent with the results of the sensitivity analysis.

## 4. Discussion 

POCD has a higher incidence after cardiovascular surgery, and it is more common in elderly patients. POCD is closely related to the long-term mortality rate after surgery. The symptoms are impairment of memory, orientation and abstract thinking of patients, as well as the ability to engage in social activities after anesthesia [28]. However, the specific mechanism of its occurrence is still unclear. Therefore, our meta-analysis comprehensively and systematically reviewed available randomized controlled trials investigating the relationship between cerebral oxygen saturation and POCD to evaluate the impact of intraoperative cerebral oxygen saturation-guided treatment on selected postoperative outcomes. The major findings of this study were that patients undergoing cardiovascular surgery who had brain oxygen saturation levels measured and maintained at high levels had a significantly lower risk of postoperative POCD than those who did not have brain oxygen saturation measurements and interventions. In addition, both cerebral oxygen testing and intervention had positive, but not statistically significant outcomes in terms of infection, hospital mortality, and stroke. We also found that patients with cardiovascular surgery who underwent intraoperative cerebral oxygen detection and intervention had a shorter ICU stay after surgery, but there was no difference in hospital stay. There was no significant difference in CPB time, mechanical ventilation time and other postoperative complications like renal failure and arrhythmia.

Previous studies have shown that there is a significant correlation between the severity of the reduction of intraoperative ${\mathrm{rScO}}_{2}$ and the incidence of postoperative neurological complications, length of hospital stay, and postoperative cognitive dysfunction [10, 29, 30]. Orihashi *et al*. [31]monitored brain oxygen saturation in 59 patients undergoing elective cerebral perfusion aortic surgery and found that 27.1% of the patients had neurological complications, including 6 patients with new cerebral infarction. The ${\mathrm{rScO}}_{2}$ decrease time and operation time of patients with complications were significantly longer than those without complications. The results also suggest that a sustained decrease in ${\mathrm{rScO}}_{2}$ during aortic surgery is closely associated with postoperative neurological complications. Schoen *et al*. [29] investigated whether ${\mathrm{rScO}}_{2}$ predicted POCD during cardiac surgery, and the results showed that POCD was associated with low ${\mathrm{rScO}}_{2}$ during cardiopulmonary bypass. Based on the above studies, we used the incidence of POCD as the primary outcome to evaluate whether timely monitoring and intervention of cerebral oxygen saturation would reduce the risk of postoperative POCD in patients undergoing cardiovascular surgery. The results of our study also coincided with the above studies. However, some studies have found that there is no correlation between ${\mathrm{rScO}}_{2}$ and POCD in heart valve surgery [32].

ICU time and hospital stay can reflect the recovery of patients after surgery, of which ICU time is more meaningful. Patients with poor prognosis will inevitably have prolonged ICU time and hospital stay. Therefore, we used ICU time and hospital stay as secondary outcomes to evaluate whether intraoperative cerebral oxygen saturation monitoring and intervention are more beneficial to patient prognosis. Adams *et al*. [23] conducted a randomized controlled study of 200 patients undergoing coronary artery bypass grafting, and found that the lower the basal and mean values of ${\mathrm{rScO}}_{2}$, the longer the postoperative ICU stay and the total postoperative hospital stay were significantly longer. Subsequently, Fisher *et al*. [5, 10] also confirmed that the reduction of ${\mathrm{rScO}}_{2}$ in cardiac surgery was positively correlated with length of stay in ICU and length of hospital stay. However, our results showed that the monitoring and intervention of intraoperative cerebral oxygen saturation resulted in shorter ICU time in patients compared with the control group, while the length of hospital stay was not significantly different between the two groups. This may be related to the different hospital management systems in different countries and regions, as well as the difference in the turnover rate of beds in different hospitals.

We initially speculated that the shorter CPB time and mechanical ventilation time could reflect the length of the operation to a certain extent, and based on this, we could evaluate whether the monitoring and intervention of intraoperative cerebral oxygen saturation were beneficial to the smooth progress of the operation. However, our results showed no significant difference between the intervention and control groups. Perhaps there is no association, so we need larger multicenter studies to verify the relationship. We also selected some common postoperative complications to verify whether intraoperative cerebral oxygen saturation monitoring and intervention would reduce the risk of postoperative complications in patients. Studies have shown that changes in cerebral oxygen saturation may be associated with postoperative renal insufficiency due to the relationship between low rSo2 values and impaired systemic tissue perfusion. This is why we chose renal failure as one of the outcomes [23, 33]. Infection is one of the common complications of various surgeries, so we were interested in exploring whether intraoperative changes in cerebral oxygen saturation were associated with the risk of postoperative infection. In addition, arrhythmia and hospital mortality can be used to assess the prognosis of patients. Stroke, as a neurological complication, can be specifically used to evaluate the postoperative neurological condition of patients. Although we found a lower risk of infection, death, and stroke in the intervention group than in the control group, these data were not statistically significant. This may be related to the size of the sample size.

At present, the pathogenesis of POCD is not clear. Studies have found that old age, years of education, preoperative comorbidities, length of anesthesia, types of surgery, preoperative medication, and postoperative infection are all risk factors for POCD [34, 35, 36]. The mechanism of intraoperative cerebral oxygen saturation monitoring and POCD reduction remains unclear, and it may be the result of multiple factors. Cardiovascular surgery is a kind of operation with a relatively long time and large trauma and high incidence of perioperative stroke. Cerebrovascular microembolism caused by plaque removal caused by surgical operation, temporary intraoperative artery blockade, and postoperative wound inflammatory reaction will all lead to arterial pressure deficiency, reduced effective cerebral perfusion, limited nerve cell function, and further lead to brain regulatory dysfunction [37]. We speculate that systemic inflammatory factors will be produced in the human body during the perioperative period, which will destroy the blood-brain barrier, and promote the infiltration of inflammatory factors and macrophages into the brain, resulting in impaired brain function. Preoperative use of dexamethasone has been found to reduce the inflammatory response, thereby reducing the risk of early POCD after cardiac surgery [13, 38]. Maintaining a high level of cerebral oxygen means that normal cerebral perfusion pressure ensures stable blood flow to the brain, thereby ensuring a continuous supply of oxygen and energy to improve cerebral vascular embolism, insufficient cerebral perfusion, tissue hypoxia, and inflammatory reactions. Oxygen metabolism imbalance thereby reducing the incidence of POCD [39]. However, the specific mechanism remains unclear, which is the direction that needs to be studied in the future.

Our study systematically investigated the impact of intraoperative cerebral oxygen saturation monitoring and intervention on cardiovascular surgery patients from intraoperative process to prognosis. It provides new directions and guidance for future research in this field. We also believe that monitoring cerebral blood perfusion during cardiac surgery is particularly necessary to reduce postoperative neurological complications, especially for elderly patients, the use of ${\mathrm{rScO}}_{2}$ monitoring can detect intraoperative cerebral ischemia and hypoxia time, predict postoperative neurocognitive function, prevent postoperative adverse events in the central nervous system, and improve the postoperative prognosis of patients. Despite the requirement for further large-scale, high-quality trials to clarify the interventions that are most effective and how they directly affect cognitive dysfunction, the results of our study suggest that a simple intraoperative procedure on the basis of cerebral oximetry may provide significant benefits.

Our meta-analysis has several limitations: First, the included studies are quite heterogeneous, and the reason may be that the source of the cases is elderly patients, and their postoperative cognitive function is affected by many factors such as diversification of surgical methods, combination of different underlying diseases, selection of different anesthetics, and so on. Due to the limited number of included literature, it is not yet possible to conduct subgroup analysis to discuss the results of different periods of postoperative POCD. Although the results of this study indicate that there are differences in the impact of intraoperative detection and intervention of cerebral oxygen saturation on patients’ cognitive impairment after surgery, there is currently a lack of large-sample and high-quality randomized controlled studies. The results of this research can provide clues and a reference basis for the design of the next research. Second, the time to evaluate POCD was relatively short (i.e., one week postoperatively), which may restrict the clinical significance of our results. However, it is worth noting that only two trials evaluated this result after three months, and both trials showed a significant reduction in POCD in the intervention group. Third, the sample size of the included randomized controlled trials is small. The lack of a sufficient number of patients to detect a meaningful difference emphasizes the necessity of further massive randomized trials.

## 5. Conclusions 

In conclusion, we conclude that maintaining intraoperative cerebral oxygen saturation at a high or stable state can significantly reduce the risk of postoperative POCD in patients with cardiovascular surgery, and can shorten the patient’s ICU time, which has a positive effect on the prognosis of the patient.

## Supplementary Material

Supplementary material associated with this article can be found, in the online version, at https://doi.org/10.31083/j.rcm2312388.
