# Correction: Wang et al. The Relationship between the Incidence of Postoperative Cognitive Dysfunction and Intraoperative Regional Cerebral Oxygen Saturation after Cardiovascular Surgery: A Systematic Review and Meta-Analysis of Randomized Controlled Trials. Reviews in Cardiovascular Medicine. 2022; 23(12): 388

**DOI:** 10.31083/RCM56196

**Published:** 2026-07-21

**Authors:** Luchen Wang, Zekun Lang, Haoyu Gao, Yanxiang Liu, Huishu Dong, Xiaogang Sun

**Affiliations:** ^1^Aortic and Vascular Surgery Center, Fuwai Hospital, National Center for Cardiovascular Diseases, Chinese Academy of Medical Sciences and Peking Union Medical College, 100037 Beijing, China; ^2^The First Clinical Medical College of Lanzhou University, 730000 Lanzhou, Gansu, China

There is a minor spelling error in the article. To ensure the accuracy and clarity of the manuscript, the authors wish to make the following corrections to this paper [1]:

On the right side of page 6, in the section “3.7 Publication Bias Assessment and Sensitivity Analysis”, line 5, the sentence “no begger test or egger test was performed” should be changed to “no Begg test or Egger test was performed”.

This correction involves only a typographical adjustment and does not affect the scientific content, data analysis, or conclusions of the original article. The authors apologize for any confusion caused by this error.
