# Supplementary material for: The Relationship between the Incidence of Postoperative Cognitive Dysfunction and Intraoperative Regional Cerebral Oxygen Saturation after Cardiovascular Surgery: A Systematic Review and Meta-Analysis of Randomized Controlled Trials
Source: Rev Cardiovasc Med. 2022 Nov 28;23(12):388. doi: 10.31083/j.rcm2312388 (PMC11270391; doi:10.31083/j.rcm2312388)
Supplement: Supplementary file 1 [file 2153-8174-23-12-388-s1.zip › 2153-8174-23-12-388-s1/search strategy.docx]

((((((Postoperative Cognitive Complications[Mesh]) OR (Postoperative Cognitive Complications[Title/Abstract])) OR (Postoperative Cognitive Complication[Title/Abstract])) OR (Postoperative Cognitive Dysfunction[Title/Abstract])) OR (Postoperative Cognitive Dysfunctions[Title/Abstract])) OR (Postoperative Decline[Title/Abstract])) OR (Postoperative Declines[Title/Abstract])

AND

(((((((((((((((((((((((((((((((Cardiovascular Diseases[Mesh]) OR (Cardiovascular Diseases[Title/Abstract])) OR (Cardiovascular Disease[Title/Abstract])) OR (Atrial Septal Defects[Title/Abstract])) OR (Atrial Septal Defect[Title/Abstract])) OR (Persistent Ostium Primum[Title/Abstract])) OR (Ostium Primum, Persistent[Title/Abstract])) OR (Atrial Septal Defect Ostium Primum[Title/Abstract])) OR (Ostium Secundum Atrial Septal Defect[Title/Abstract])) OR (Ventricular Septal Defect[Title/Abstract])) OR (Ventricular Septal Defects[Title/Abstract])) OR (Intraventricular Septal Defects[Title/Abstract])) OR (Intraventricular Septal Defect[Title/Abstract])) OR (Aortic Coarctation[Title/Abstract])) OR (Aortic Coarctations[Title/Abstract])) OR (Coarctation of the Aorta[Title/Abstract])) OR (Coarctation of Aorta[Title/Abstract])) OR (Aorta Coarctation[Title/Abstract])) OR (Aorta Coarctations[Title/Abstract])) OR (Coarctation of Aorta Dominant[Title/Abstract])) OR (Aorta Dominant Coarctation[Title/Abstract])) OR (Aorta Dominant Coarctations[Title/Abstract])) OR (Coronary Artery Disease[Title/Abstract])) OR (Coronary Artery Diseases[Title/Abstract])) OR (Mitral Valve Insufficiency[Title/Abstract])) OR (Mitral Valve Regurgitation[Title/Abstract])) OR (Mitral Regurgitation[Title/Abstract])) OR (Mitral Valve Incompetence[Title/Abstract])) OR (Mitral Incompetence[Title/Abstract])) OR (Aortic Valve Stenosis[Title/Abstract])) OR (Aortic Valve Stenoses[Title/Abstract])) OR (Aortic Stenosis[Title/Abstract])

AND

(((((regional cerebral oxygen saturation[Title/Abstract]) OR (rScO2[Title/Abstract])) OR (cerebral regional oxygen saturation[Title/Abstract])) OR (cerebral oxygen saturation[Title/Abstract])) OR (saturation of cerebral blood oxygen[Title/Abstract])) OR (oxygen saturation[Title/Abstract])
