# Supplementary material for: The Relationship between the Incidence of Postoperative Cognitive Dysfunction and Intraoperative Regional Cerebral Oxygen Saturation after Cardiovascular Surgery: A Systematic Review and Meta-Analysis of Randomized Controlled Trials
Source: Rev Cardiovasc Med. 2022 Nov 28;23(12):388. doi: 10.31083/j.rcm2312388 (PMC11270391; doi:10.31083/j.rcm2312388)
Supplement: Supplementary file 1 [file 2153-8174-23-12-388-s1.zip › 2153-8174-23-12-388-s1/SM Table1 PICOS.docx]

| PICOS |  |
| --- | --- |
| 1.Participants | Patients undergoing cardiovascular surgery. |
| 2.Intervention | Carry out rScO_2_ detection and take corresponding measures to improve rScO_2_. |
| 3.Comparison | No rScO_2_ detection and processing under blinding. |
| 4.Outcomes | POCD incidence, Stroke, Atrial fibrillation, Renal failure, ICU time, Operation time, infection, etc. |
| 5.Study design | Randomized controlled study. |

Tabel 1 of supplementary materials "PICOS" approach for selecting clinical studies in the systematic search.
