# Supplementary figures and images for: The Relationship between the Incidence of Postoperative Cognitive Dysfunction and Intraoperative Regional Cerebral Oxygen Saturation after Cardiovascular Surgery: A Systematic Review and Meta-Analysis of Randomized Controlled Trials
Source: Rev Cardiovasc Med. 2022 Nov 28;23(12):388. doi: 10.31083/j.rcm2312388 (PMC11270391; doi:10.31083/j.rcm2312388)

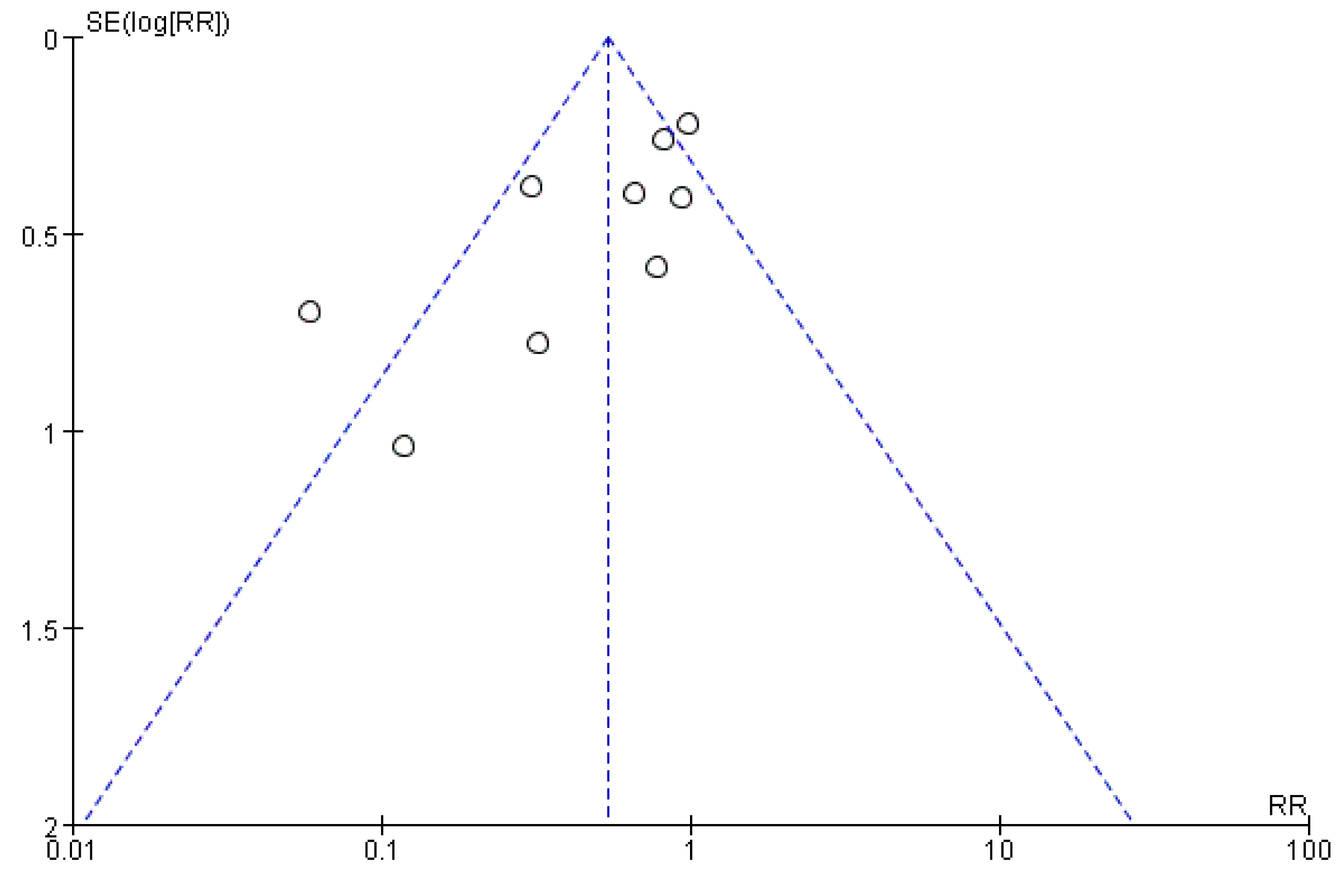

Supplement: Supplementary file 1 [file 2153-8174-23-12-388-s1.zip › 2153-8174-23-12-388-s1/Funnel plot of POCD incidence.jpg]

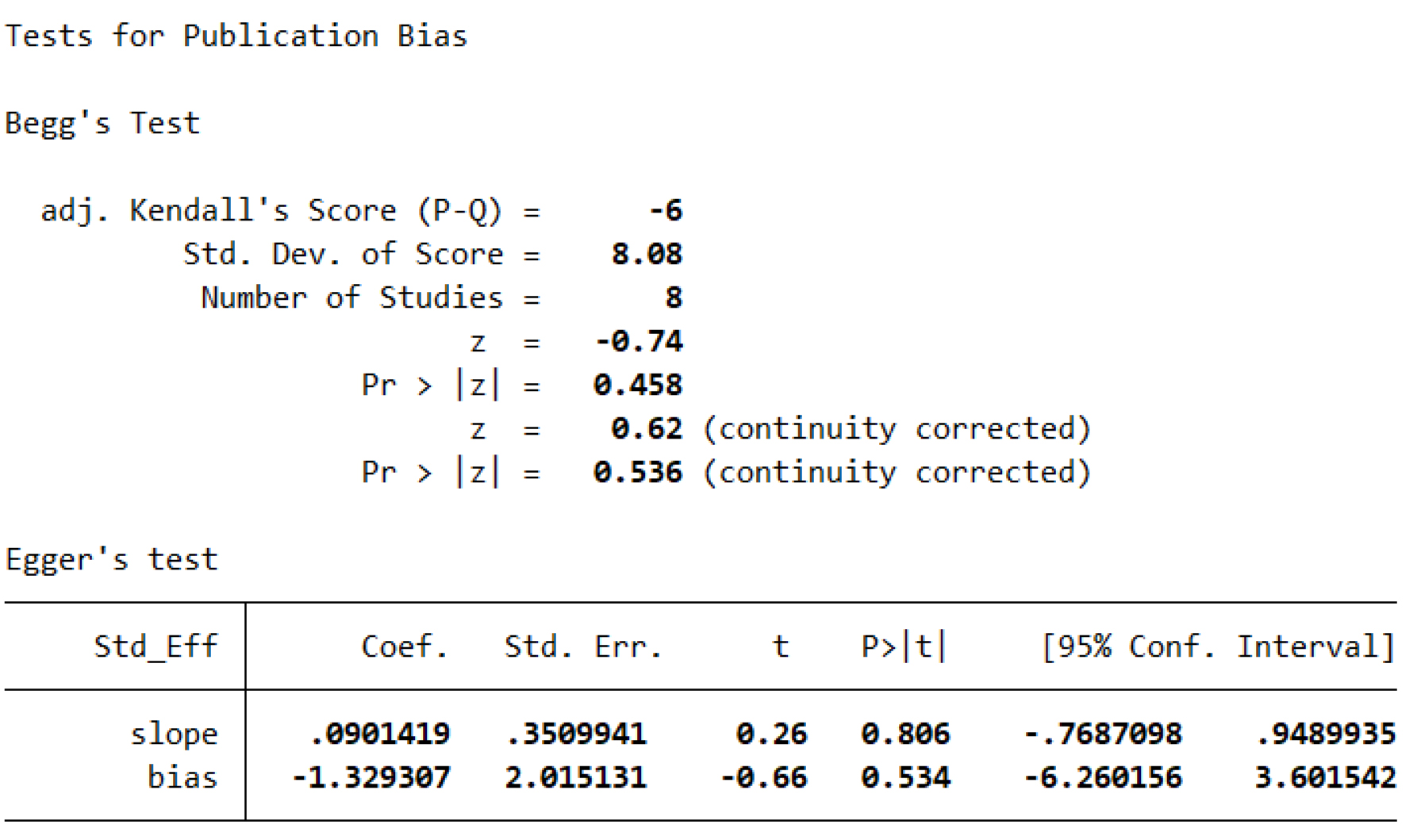

Supplement: Supplementary file 1 [file 2153-8174-23-12-388-s1.zip › 2153-8174-23-12-388-s1/publication bias of ICU time.jpg]

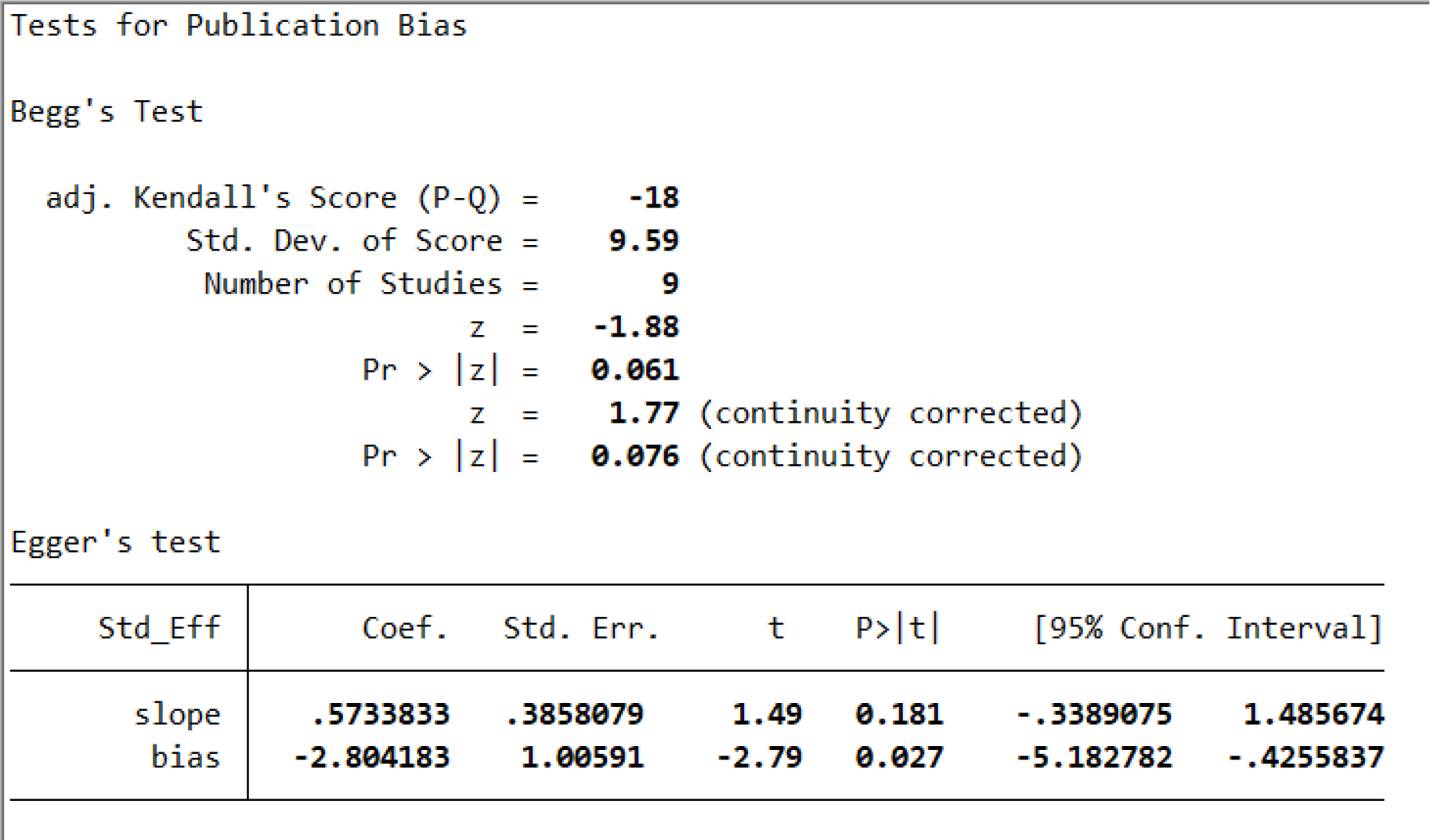

Supplement: Supplementary file 1 [file 2153-8174-23-12-388-s1.zip › 2153-8174-23-12-388-s1/publication bias of POCD incidence.jpg]

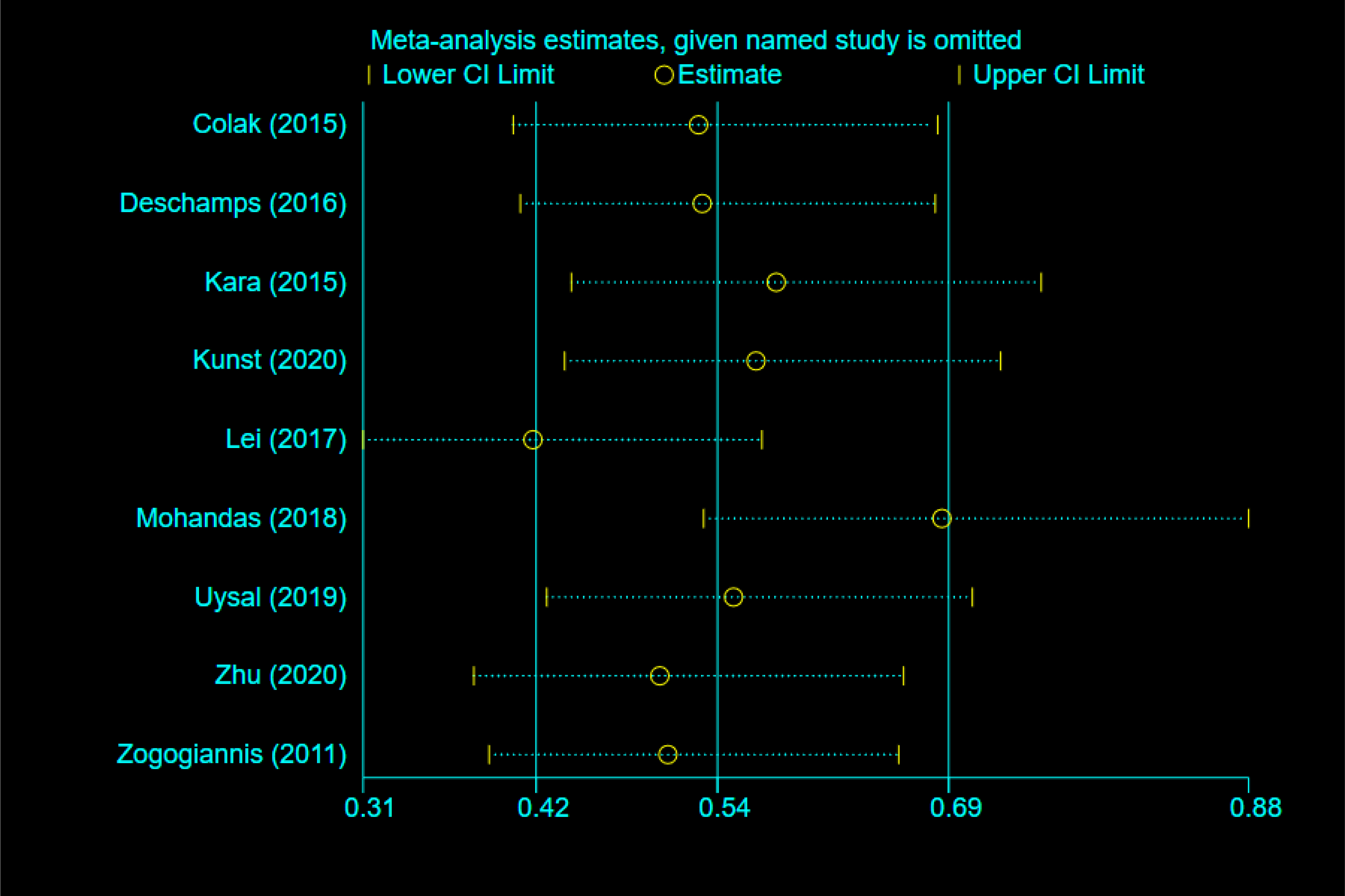

Supplement: Supplementary file 1 [file 2153-8174-23-12-388-s1.zip › 2153-8174-23-12-388-s1/Sensitivity analysis of POCD incidence.jpg]
